# Validez y confiabilidad del Beliefs About Medicines Questionnaire en pacientes hipertensos colombianos

**DOI:** 10.15649/cuidarte.1937

**Published:** 2021-08-20

**Authors:** Eugenia del Pilar Herrera Guerra, Juana Raquel Robles González, Lili Rosa Bautista Arellano

**Affiliations:** 1 Universidad de Córdoba, Facultad de Ciencias de la Salud, Enfermera, Montería, Colombia. Email: edherrera@correo.unicordoba.edu.co Autor de correspondencia Universidad de Córdoba Universidad de Córdoba Facultad de Ciencias de la Salud Montería Colombia edherrera@correo.unicordoba.edu.co; 2 Universidad de Córdoba. Facultad de Ciencias Básicas, Estadística, Montería, Colombia. Email: jrobles@correo.unicordoba.edu.co Universidad de Córdoba Universidad de Córdoba Montería Colombia jrobles@correo.unicordoba.edu.co; 3 Universidad de Córdoba. Facultad de Ciencias Básicas, Estadística, Montería, Colombia. Email: lbautistaarellano72@correo.unicordoba.edu.co Universidad de Córdoba Universidad de Córdoba Facultad de Ciencias Básicas Montería Colombia lbautistaarellano72@correo.unicordoba.edu.co

**Keywords:** Hipertensión Esencial, Enfermedades Cardiovas- culares, Creencias, Psicometría, Estudios de validación., Essential Hypertension, Cardiovascular Diseases, Beliefs, Psychometrics, Validation Study., Hipertensão Essencial, Doenças Cardiovasculares, Crenças, Psicometria, Estudos de validação.

## Abstract

**Introducción.:**

El *Beliefs about Medicines Questionnaire* (*BMQ*) permite valorar las representaciones cognitivas que engloban las creencias sobre la medicación de los pacientes, sobre tomar medicamentos para su enfermedad en diferentes culturas.

**Objetivo.:**

Determinar la validez de constructo y confiabilidad del cuestionario *BMQ* adaptado a pacientes hipertensos colombianos.

**Materiales y métodos.:**

Estudio psicométrico de tipo instrumental, realizado en una muestra de 238 pacientes hipertensos en edad promedio de 65 años (*DE=* 11,4) con predominio del sexo femenino (70%). La validez de constructo se evaluó mediante Análisis Factorial Exploratorio y Confirmatorio*.* Se calculó la confiabilidad utilizando el método coeficiente de alfa de Cronbach.

**Resultados.:**

Se obtuvo una versión reducida de 16 ítems; en la sección *BMQ*- General los 7 ítems se agruparon en dos factores que explicó el 64% de la varianza común y buen ajuste (χ^2^= 61.46; *gl* = 13; *p* = 0.000; CFI = 0.917; NNFI = 0.89; CFI=0.917; SRMR=0.054; RMSEA = 0.125; IC 90% [0,10, 0,16]). En el *BMQ*-Específico los 9 ítems agrupados en dos factores que explicaron el 63,17% de la varianza común con un ajuste aceptable (χ^2^= 122.4; *gl* = 26; *p* = 0.000; CFI = 0.88; NNFI = 0.84; CFI=0.88; SRMR=0.106; RMSEA = 0.125; IC 90% [0.10, 0.15]). La confiabilidad por alfa de Cronbach para el *BMQ*- General y Específico fue de 0.82 y 0.78 respectivamente.

**Discusión y conclusiones.:**

La versión del *BMQ* adaptada a pacientes hipertensos colombianos, poseen características psicométricas adecuadas, su uso es recomendado en la investigación.

## Abstract (en)

**Introduction.:**

The Beliefs about Medicines Questionnaire (BMQ) allows the assessment of cognitive representations encompassing patients’ medication beliefs about taking medication for their disease in different cultures.

**Objective.:**

To determine the construct validity and reliability of the BMQ questionnaire adapted to Colombian hypertensive patients.

**Materials and methods.:**

An instrumental psychometric study was carried out in a sample of 238 hypertensive patients with an average age of 65 years (SD= 11.4), with a predominance of women (70%). Construct validity was assessed by exploratory and confirmatory factor analysis. Reliability was calculated using Cronbach’s alpha coefficient method.

**Results.:**

A reduced version of 16 items was obtained; in the BMQ-General section the 7 items were grouped into two factors that explained 64% of the common variance and good fit (χ^2^= 61.46; *gl* = 13; *p* = 0.000; CFI = 0.917; NNFI = 0.89; CFI=0.917; SRMR=0.054; RMSEA = 0.125; IC 90% [0,10, 0,16]). In the BMQ-Specific the 9 items grouped into two factors explained 63.17% of the common variance with an acceptable fit (χ^2^= 122.4; *gl* = 26; *p* = 0.000; CFI = 0.88; NNFI = 0.84; CFI=0.88; SRMR=0.106; RMSEA = 0.125; IC 90% [0.10, 0.15]). The Cronbach’s alpha reliability for the BMQ-General and Specific was 0.82 and 0.78, respectively.

**Discussion and conclusions.:**

The version of the BMQ adapted to Colombian hypertensive patients has adequate psychometric characteristics and its use is recommended in research.

## Resumo

**Introdução.:**

O Beliefs About Nedicines Questionnaire (BMQ) permite a avaliação de representações cognitivas abrangendo as crenças dos pacientes sobre os de medicamentos e seu uso para suas doenças em diferentes culturas.

**Objetivo.:**

Determinar a validade de constructo e a confiabilidade do questionário BMQ adaptado a pacientes hipertensos colombianos.

**Materiais e métodos.:**

Foi realizado um estudo psicométrico instrumental em uma amostra de 238 pacientes hipertensos com idade média de 65 anos (DE = 11,4), com predominância de mulheres (70%). A validade de constructo foi avaliada mediante Análise Fatorial Exploratória e Confirmatória de Fatores. A confiabilidade foi calculada usando o método do coeficiente alfa de Cronbach.

**Resultados.:**

Uma versão reduzida de 16 itens foi obtida; na seção BMQ-General os 7 itens foram agrupados em dois fatores que explicaram 64% da variância comum e bom ajuste (χ^2^= 61.46; *gl* = 13; *p* = 0.000; CFI = 0.917; NNFI = 0.89; CFI=0.917; SRMR=0.054; RMSEA = 0.125; IC 90% [0,10, 0,16]). No BMQ-Específico os 9 itens agrupados em dois fatores explicaram 63,17% da variância comum com um ajuste aceitável (χ^2^= 122.4; *gl* = 26; *p* = 0.000; CFI = 0.88; NNFI = 0.84; CFI=0.88; SRMR=0.106; RMSEA = 0.125; IC 90% [0.10, 0.15]). A confiabilidade alfa de Cronbach para o BMQ-General e Específico foi de 0,82 e 0,78 respectivamente.

**Discussão e conclusões.:**

A versão do BMQ adaptada aos pacientes hipertensos colombianos tem características psicométricas adequadas e seu uso é recomendado na pesquisa.

## Introducción

La hipertensión arterial (HTA) es una de las principales causas de morbimortalidad cardiovascular en el mundo(1). En Colombia la HTA es la tercera causa de mortalidad por enfermedades del sistema circulatorio(2). Dentro de las explicaciones posibles de esta problemática se encuentran fenómenos poblacionales como la diversidad de razas, costumbres y nivel sociocultural variado, sumado a otros factores que aumentan el riesgo cardiovascular y afectan la adherencia al tratamiento(3). Al respecto, los estudios revelan que la adherencia al tratamiento de la HTA está mediada, entre otros factores, por las creencias que el paciente tenga sobre su tratamiento(4),(5).

Los pacientes con HTA han referido su experiencia al tener que tomar pastillas el resto de su vida, manifestando fuertes creencias sobre posibles efectos adversos, temores a la posible interacción con otros tratamientos, sentimientos de peligro a la dependencia y a la toxicidad a largo plazo; creencias que pueden reducir la adherencia al tratamiento(6),(7),(8).

El constructo necesidad y preocupaciones por la medicación proporciona un marco simple para operacionalizar creencias específicas relacionadas con la adherencia al tratamiento, dentro del contexto de las teorías de la cognición social y la autorregulación(9). Se ha reconocido que la mayoría de los pacientes suelen construir modelos mentales que engloban sus creencias sobre los medicamentos en general y sobre el tratamiento específico de su enfermedad. Así mismo, la mayoría de las personas elabora esquemas mentales, que, en muchos casos, contienen información bastante negativa sobre rasgos generales asociados a los medicamentos. Del mismo modo, las personas elaboran una visión personal de la necesidad de tomar su medicación, así como de los aspectos negativos de tomarla que pueden generarles cierto nivel de preocupación(10). De allí la importancia de la valoración de las creencias sobre la medicación en los pacientes con enfermedades crónicas como la HTA.

El cuestionario *Beliefs about Medicines Questionnaire (BMQ*)(11) permite evaluar las creencias sobre la medicación entendida como representaciones cognitivas de los pacientes sobre la medicación en general (*BMQ*-General) y sobre la medicación para su tratamiento específico (*BMQ*-Específico) en diferentes enfermedades crónicas. El *BMQ* ha sido traducido del idioma original (ingles) a varios idiomas y validado en pacientes con enfermedades crónicas como la artritis reumatoidea(12), diabetes(13), enfermedad pulmonar obstructiva crónica(14), asma(15), accidente cerebrovascular(16), enfermedad mental(17), VIH(18), HTA(19), en otras patologías(20),(21),(22),(23), en diferentes países. Los estudios psicométricos proporcionan evidencia satisfactoria de validez y confiabilidad. La versión del *BMQ* en español ha sido validada en pacientes psiquiátricos(24), con asma(25) y con diabetes e HTA(26).

El *BMQ* en español fue adaptado y validado en España por Beléndez, et al(24); para validar la sección *BMQ*-General, tomaron pacientes crónicos (n=156) y estudiantes universitarios (n= 256). El *BMQ*- Específico se estudió en 97 pacientes diabéticos tratados con insulina y 59 hipertensos tratados con antihipertensivos. La versión fue revisada aprobada por el autor de la versión original del *BMQ*. Los resultados comprobaron la estructura bifactorial del modelo teórico de la versión original para las dos subsecciones. En el análisis factorial exploratorio del *BMQ-*General en el grupo de pacientes crónicos (*n* = 156) resultó una estructura bifactorial que explicaba el 53% de la varianza total. En el *BMQ*-Específico para pacientes hipertensos resultó una estructura de dos factores que explicaron un 55% de la varianza total. Los índices de consistencia interna para el factor necesidad fue 0,47 y para el factor preocupación fue 0,35. Los resultados que apoyan la validez de constructo. Los autores recomiendan realizar otros estudios en pacientes con HTA por el reducido tamaño de la muestra (*n* =59).

Los estudios psicométricos del *BMQ* versión original proporcionan evidencias de validez y confiabilidad, siendo considerado una medida útil para la evaluación de las creencias sobre la medicación. Sin embargo, hasta el momento, existe escasa evidencia empírica de las propiedades psicométricas del *BMQ* versión español en pacientes hipertensos Latinoamericanos. Cabe resaltar que la literatura reporta que la versión española del *BMQ*-General fue utilizada en Bogotá-Colombia en una muestra de pacientes hipertenso. No obstante; el estudio no utilizó la sección *BMQ*-Especifico ni proporciona evidencia psicométrica, por lo que se hace necesario realizar estudios adicionales. Por tanto, se decidió realizar el presente estudio que tuvo como objetivo determinar la validez de constructo y confiabilidad del cuestionario *BMQ* versión español en pacientes hipertensos colombianos.

## Materiales y métodos

Se realizó un estudio de tipo metodológico(28). Participaron 238 pacientes hipertensos inscritos en programas de control de la HTA en 4 centros de salud de la red de atención de primer nivel en Montería- Colombia, en el segundo semestre de 2019. Para la determinación del tamaño muestral se fijó un número superior a 200 y una tasa superior a 10 sujetos por variable, recomendado para los análisis psicométricos, para ofrecer buenas garantías en la estimación de los parámetros, especialmente con modelos que incluyen pocas variables(29), como ocurre en este estudio. De una población de 620 se calculó la muestra con un nivel de confianza 95%, *p* = 0.5, *q* = 0.5 y un margen de error del 5%. Los participantes fueron, seleccionados al azar por muestreo estratificado. El proceso de selección de la muestra fue aleatorio sistemático. Los criterios de inclusión fueron ser mayor de 18 años, estar diagnosticado con HTA y tener prescrita medicación antihipertensiva durante al menos 6 meses al momento de la encuesta. Se excluyeron pacientes con comorbilidad alta, déficit mental y/o sensorial.

Instrumentos. Se utilizó cuestionario *BMQ* versión español adaptada para pacientes hipertensos por Beléndez, et al(24), compuesto por dos secciones, el *BMQ*-General (8 ítems) que incluye dos factores: abuso (4 ítems) y daño (4 ítems) y *BMQ*-Específico (10 ítems) donde el término medicamento fue cambiando por pastillas; incluye dos factores: necesidad (5 ítems) y preocupación (5 ítems). Todos los ítems se evalúan mediante una escala de cinco puntos desde 1 (totalmente en desacuerdo) hasta 5 (totalmente de acuerdo). Las puntuaciones más altas indican fuertes creencias negativas sobre los conceptos evaluados en cada subescala; es decir sobre daño y en abuso para todos los medicamentos y sobre la percepción de usar medicamentos específicos para mantener la salud en comparación con la necesidad personal y una mayor preocupación por sobre los efectos adversos del consumo de medicamentos. Las dos secciones del *BMQ* se pueden utilizar en combinación o por separado. También se aplicó un cuestionario de datos sociodemográficos y clínicos.

Procedimiento. Un traductor oficial y un lingüista evaluaron la equivalencia semántica, idiomática, conceptual y cultural del *BMQ* versión español adaptada para pacientes hipertensos por Beléndez, et al(24). Seguidamente tres jueces expertos con formación de doctorado y experiencia en psicometría revisaron el cuestionario. El 100% estuvo de acuerdo en no hacer cambios a los ítems. Seguidamente se realizó una prueba piloto en un grupo de adultos (n=50) en edades de 48 a 87 años para revisar la comprensión de los ítems, como resultado no hubo cambios en la redacción de los ítems. Los pacientes hipertensos completaron los instrumentos durante su cita de control, previo consentimiento informado individual escrito. Los datos se recogieron de agosto a octubre de 2019, con la participación de dos estudiantes de enfermería capacitados para tal fin.

Los datos se analizaron en el programa estadístico SPSS versión 22.0. Inicialmente, se realizó un análisis de los descriptivos (media, desviación estándar, asimetría, curtosis y coeficiente de correlación corregido ítem-total), esperando obtener cálculos de los índices de asimetría y curtosis, entre ± 1.96 en el examen de normalidad(30). Para evaluar la adecuación del tamaño de la muestra y la correlación entre las variables, necesaria para establecer la factibilidad del análisis factorial se usó el estadístico *Kaiser-Meyer-Olkin* (*KMO*) y la prueba de esfericidad de Bartlett. En este estudio, se consideró un punto de inflexión de 0.4 como el mínimo de factor de carga requerido para mantener cada elemento extraído del análisis factorial.

El análisis factorial exploratorio (AFE) se realizó mediante el método de componentes principales y rotación con Varimax *Kaiser.* El análisis factorial confirmatorio (AFC) se hizo el programa estadístico IBM SPSS Amos - versión 26.0. Se utilizó el análisis de máxima verosimilitud para medir la validez de estructura y conformidad de los ítems de la versión española del cuestionario con la versión colombiana. Previamente al análisis de estimación de los modelos, se calculó el coeficiente de *Mardia*, para asegurar la presencia de normalidad multivariada en los datos obtenidos, en el que valores menores a 70 se consideran indicativos de normalidad multivariada de los datos(31). Para determinar la bondad del ajuste se calcularon la prueba Chi cuadrado (χ^2^), (no significativas, *p* > 0,05), el índice de Kaike (*Akaike Index-*AIC), índice de ajuste comparativo *Comparative Fit Index* - CFI) y el índice de ajuste no normado (*Non Normed Fit Index* -NNFI) para valores mayores que 0.95 o superiores son considerados excelentes, y valores superiores a 0,90 sugieren un ajuste aceptable del modelo a los datos. Entre los índices basados en las covarianzas se tomó el residual cuadrático medio estandarizado *(Standardized Mean Square Residual* - SRMS) y el error cuadrático medio de aproximación *(Root Mean Square Error of Approximation* - RMSEA). Se considera que RMSEA < 0,05 indica un buen ajuste a los datos si, además, el intervalo de confianza al 90% se sitúa entre 0 y 0,05 y ajuste aceptable en el rango 0,05-0,08.

La confiabilidad se evaluó calculando la consistencia interna de cada subescala y factor a través del índice alfa de Cronbach. Se espera un valor igual o superior a 0.70 para considerar que el *BMQ* es confiable para usar en investigación(32).

### Aspectos éticos

Esta investigación adoptó las consideraciones éticas reglamentadas por la Resolución 8430 de 1993(33). El estudio se clasificó como investigación sin riesgo y fue aprobado por el Comité de Ética de la Facultad Ciencias de la Salud de la Universidad de Córdoba según acta de 13 de mayo de 2019. Se obtuvo el aval institucional; todos los pacientes firmaron consentimiento informado escrito.

## Resultados

Un total de 238 pacientes hipertensos fueron reclutados en este estudio, seleccionados al azar. La mayoría eran mujeres (70%), la edad promedio fue de 65 años (*DE=* 11,4), la mayoría reportaron bajo nivel educativo (82%) y bajos ingresos económicos (82%); el tiempo promedio de estar diagnosticado con HTA fue de 8 ± 7,66 años.

### Validez de constructo

El índice de adecuación muestral *Kaiser-Meyer-Olkin* del *BMQ*-General (KMO=0,782; χ^2^= 597,472; *gl=* 21; p < 0,000.) presentó un valor adecuado para la aplicación del análisis factorial. Así mismo para el *BMQ*-Específico (KMO=0,786; χ^2^= 854,74; *gl=36*; *p* < 0,000.) lo que indica que la matriz de correlaciones y la matriz de identidad presentan diferencias, es decir, se evidencia correlación entre las variables. Se corrió el AFE para el *BMQ*-General únicamente con los 7 ítems que mostraban una correlación ítem total corregida igual o superior a 0,30, lo que implicaba la eliminación del ítem 7 (Los médicos confían demasiado en los medicamentos). Las comunalidades oscilaron entre 0,60 y 0,74 para estos 7 ítems. Por otro lado, al realizar al AFE del *BMQ*-Especifico con los 10 ítems; el ítem 2 (Me preocupa tener que tomar medicamentos) y 3 (Mi vida sería imposible sin los medicamentos) se asociaron, sin embargo, no constituyeron un factor al no tener coherencia teórica ni el número de ítems para determinar una dimensión. Se observó que el ítem 3 compartía carga factorial mayor que 0.4 en un segundo factor. Por tanto, fue eliminado el ítem 2. Las comunalidades oscilaron entre 0.50 y 0.76 para la versión de 9 ítems.

La Tabla 1 muestra el AFE de las dos secciones del *BMQ*. Los resultados en la sección *BMQ*- General sugieren retener dos factores: daño que explicó el 35,3% de la varianza y abuso que explicó el 28,8% de la varianza. Los dos factores explicaron el 64% de varianza común. El factor daño quedó conformado por 3 ítems (2, 5 y 6) con las mayores cargas factoriales y el factor abuso quedo conformado por 4 ítems (1, 3, 4 y 8). La distribución de los ítems es similar al modelo teórico original, con excepción del ítem 3 (La mayoría de medicamentos crean adicción) que se incluyó en el factor abuso.

En la sección *BMQ*- Específico los resultados sugieren retener dos factores: necesidad que explicó el 36,2% de la varianza y preocupación que explicó el 26,9% de la varianza. Los dos factores explicaron el 63% de varianza común. El factor necesidad quedó conformada por 5 ítems (1, 3, 5, 7 y 10) y el factor 2 preocupación por 4 ítems (4, 6, 8 y 9). La distribución de los ítems es similar al modelo teórico original. Los ítems con mayor importancia en la medición fueron los del factor necesidad con las mayores cargas factoriales; indicando que por el contexto cultural de la muestra los componentes abuso y preocupación del cuestionario pudiera ser reformulado.

**Tabla 1:** Cargas factoriales del BMQ en pacientes hipertensos colombianos.

BMQ- General	Factor	
Estructura bidimensional	1 (Daño)	2 (abuso)
5. Los medicamentos hacen más mal que bien	0,816	
2. La gente que toma medicamentos debería dejar su tratamiento durante algún tiempo de vez en cuando	0,802	
6. Todos los medicamentos son venenos (tóxicos)	0,784	
8. Si los médicos tuvieran más tiempo para los pacientes recetarían menos medicamentos		0,772
4. Los remedios naturales son más seguros que los medicamentos		0,767
1. Los médicos utilizan demasiados medicamentos		0,649
3. La mayoría de medicamentos crean adicción		0,552
%Varianza	35,328	28,822
%Varianza acumulada	35,328	64,150
KMO = 0,782; χ^2^=597,472; *gl*= 21; *p* < 0,000			
BMQ - Específico	Factor	
Estructura bidimensional	1 (Necesidad)	2 (Preocupación)
5. Sin pastillas estaría muy enfermo/a	0,847	
1. Actualmente mi salud depende de las pastillas	0,819	
7. En el futuro mi salud dependerá de las pastillas	0,789	
10. Las pastillas impiden que mi hipertensión empeore	0,763	
3. Mi vida sería imposible sin las pastillas	0,724	
8. Las pastillas trastornan mi vida		0,862
6. Las pastillas son un misterio para mí		0,838
9. A veces me preocupo por si llego a ser demasiado dependiente de las pastillas		0,698
4. A veces me preocupo por los efectos a largo plazo de las pastillas		0,655
%Varianza	36,247	26,923
%Varianza acumulada	36,247	63,170
KMO= 0,786; =854,74; *gl*= 36; *p* < 0,000			

Fuente: Autoría propia. Método de extracción: análisis de componentes principales. Se aceptaron cargas > 0,40. Método de rotación: Normalización Varimax con Kaiser.

Con el resultado bifactorial obtenidos en el AFE se procedió al cálculo del AFC. Se comprobó la existencia de normalidad multivariada en los datos obtenidos, encontrando que los índices de asimetría y curtosis, registrados en la Tabla 2, son próximos al valor cero y por debajo del valor 1.96; De igual modo, se comprobó la normalidad multivariada de los datos a través del coeficiente de *Mardia* para los ítems en *BMQ*-General y Específico que fue de 12.643 y 24.775, respectivamente.

La Tabla 3 muestra los índices de ajuste para el modelo del *BMQ*-General, en el cual los índices estuvieron dentro de los parámetros esperados indicando un buen ajuste del modelo en cuestión. Para el modelo del *BMQ*-Específico los índices de ajuste fueron aceptables al encontrarse en un valor cercano de los parámetros esperados.

**Tabla 2:** Análisis descriptivo de los ítems en el BMQ en pacientes hipertensos colombianos.

	M ± DE	Asimetría	Curtosis	Correlación total	Alfa de Cronbach si el item es suprimido
BMQ -General					
1	3,18 ± 1,014	0,055	-0,747	0,606	0,726
2	2,51 ± 0,948	0,593	-0,091	0,452	0,753
3	2,90 ± 0,999	0,276	-0,481	0,615	0,725
4	3,15 ± 1,109	0,181	-0,922	0,572	0,731
5	2,60 ± 0,977	0,426	-0,234	0,637	0,721
6	2,40 ± 0,933	0,622	0,148	0,528	0,741
7	3,69 ± 1,031	-0,617	-0,115	0,032	0,819
8	3,07 ± 1,094	0,347	-0,556	0,424	0,758
BMQ Especifico					
1	3,18 ± 1,014	-0,388	0,058	0,451	0,757
2	3,35 ± 1,097	-0,388	-0,605	0,347	0,770
3	3,15 ± 1,109	-0,388	-0,362	0,535	0,745
4	3,62 ± 0,936	-0,388	-0,730	0,491	0,751
5	3,69 ± 1,031	-0,388	-0,091	0,529	0,748
6	3,54 ± 0,977	0,592	-0,262	0,336	0,772
7	3,07 ± 1,094	-0,326	-0,303	0,596	0,739
8	3,79 ± 0,903	0,671	-0,201	0,310	0,776
9	2,95 ± 1,046	-0,159	-0,795	0,442	0,758
10	3,56 ± 0,924	-0,286	-0,431	0,434	0,760

Fuente: Autoría propia. M: Media, DT = Desviación típica, Asimetría, Curtosis, Correlación ítem-total, Alfa de Cronbach si el ítem es suprimido.

**Tabla 3:** Análisis factorial confirmatorio escala BMQ en pacientes hipertensos colombianos.

Modelo	𝝌2	gl	p	AIC	NNFI	CFI	SRMR	RMSEA	IC 90%
BMQ - General									
Dos factores segeridos por AFE	61,46 *	13	0,000	91,460	0,899	0,917	0,054	0,125	0,095 - 0,157
BMQ - Específico									
Dos factores segeridos por AFE	122,40*	26	0,000	160,400	0,840	0,884	0,106	0,125	0,103 - 0,148

Fuente: Autoría propia. IC: intervalo de confianza: *2*: chi-cuadrado; *gl*: grados de libertad; *significancia *p* > 0.05; AIC: *Akaike Index*; NNFI: *Non-Normed of Fit Index*; CFI: *Comparative Fit Index;* SRMR: *Standard Residual Mean Root*; RMSEA: Root *Mean Square Error of Approximation*.

### Confiabilidad

En la Tabla 4 se muestra que el *BMQ* versión español adaptado a pacientes hipertensos colombianos posee un nivel de consistencia interna aceptable. Cabe resaltar que el alfa de Cronbach calculado para el *BMQ*-General (α = 0,77) y para el factor abuso (α = 0,59) aumentó al eliminar el ítem 7 tanto en el *BMQ*-General (α = 0,82) como en el factor abuso (α = 0,74). Teniendo en cuenta el análisis de los resultados del alfa de Cronbach del BMQ-General (Ver Tabla 2), el valor de alfa aumentó sensiblemente al eliminar el ítem 7 (Los médicos confían demasiado en los medicamentos). En virtud de lo anterior y teniendo en cuenta que el índice de homogeneidades (Correlación total de elemento corregida) es menor que se justifica la eliminación de dicho ítem. Respecto la consistencia interna del *BMQ*-Específico la consistencia interna resultó aceptable (α = 0,78). El alfa de Cronbach arrojó un valor de 0,85 para el factor necesidad y 0,72 para el factor preocupación que aumento a 0,77 al eliminar el ítem 2 (Me preocupa tener que tomar medicamentos).

**Tabla 4:** Coeficientes de confiabilidad alfa de Cronbach y correlación ítem-total, de los componentes del BMQ en pacientes Hipertensos colombianos

Componentes BMQ			
	alfa de Cronbach (𝛼)	Rho
BMQ General	0,82	0,669*
General abuso	0,74	0,833*
General daño	0,79	0,874*
BMQ Específico	0,78	0,728*
Específico necesidad	0,85	0,749*
Específico preocupación	0,72	0,722*

Fuente: Fuente: Autoría propia.

* Coeficiente de correlación Rho de *Sperman*.

## Discusión

Los resultados del análisis de validez de constructo del *BMQ* versión español adoptada en pacientes colombianos con HTA corroboran que la estructura factorial de la versión original y de la versión en español; basado en un modelo de cuatro factores se obtuvo una versión simplificada con un total de 16 ítems. El AFC demostró un ajuste aceptable del modelo bifactorial del *BMQ-* General y Especifico. Es importante considerar que el modelo no cumple con los requisitos (CFI ≥ 0,95 y RMSEA ≤ .05 (90% I.C, 0,00 - 0.05) por tanto, se presentaron los índices de ajuste junto con el χ2 del modelo propuesto, sus grados de libertad y la probabilidad asociada.

Los resultados del AFE *BMQ-*General sugirieron cambios frente a la distribución de los ítems 5 (Los medicamentos hacen más mal que bien) y 6 (Todas los medicamentos son veneno -tóxicos) que corresponden al factor daño se incluyeron en el factor abuso. No obstante los autores de la de la versión en español también encontraron algunas diferencias relacionadas con la ubicación de un ítem en el factor daño en vez de abuso(24). Las características intrínsecas de los participantes pueden ser relevantes para explicar la distribución de los ítems.

El presente análisis, consideró modificaciones al *BMQ*- General haberse eliminando el ítem 7 (Los médicos confían demasiado en los medicamentos) y al *BMQ*-Específico al haberse eliminado el ítem 2 (Me preocupa tener que tomar medicamentos) que presentaron bajas correlaciones y una reducción en alfa de Cronbach. Sin embargo, se hallaron las ligeras ganancias con estas modificaciones potenciales frente a otras versiones del cuestionario. Los resultados brindan un buen apoyo para confirmar la confiabilidad del *BMQ*-Especifico adaptado para pacientes hipertensos, los alfa de Cronbach para los factores necesidad (α = 0,85) y preocupación (α = 0,72) fueron superiores para necesidad y similares para preocupación a los informados en la versión original en inglés, en la que al alfa osciló entre 0,55 y 0,86 para las subescalas de necesidad y entre 0,63 a 0,80 preocupación(9), así como con los resultados obtenidos para versión en italiano (necesidad α = 0,78; preocupación α = 0,72)(34), en alemán (α = 0.83 para ambas secciones)(35), en portugués (necesidad α = 0,75; preocupación α = 0,78)(36), en polaco (necesidad α = 0,64; preocupación α = 0,82)(37) y en malayo (necesidad α = 0,76 y 0,62**¸**preocupación α = 0,62)(38).

El metaanálisis realizado por Horne R, et al(39) confirmó que el *BMQ* es un cuestionario que tiene validez predictiva para predecir la adherencia. El estudio reveló que la mayor adherencia al tratamiento se asocia con percepciones más altas de necesidad especifica del tratamiento (OR = 1,742, IC 95% [1,569; 1,934], p < 0,0001) y menos preocupaciones específicas sobre el tratamiento (OR = 0,504, IC 95%: [0,450; 0,564], p < 0,0001). Así mismo en el metaanálisis realizado por Nie B, et al se encontró que la adherencia se correlacionó significativamente con las creencias específicas de necesidad (coeficiente de correlación combinado = 0,32, IC del 95%: 0,21- 0,43), con las preocupaciones (−0,35, IC del 95%: −0,42, −0,28) y con la puntuación diferencial de preocupaciones y necesidades (0,25, IC 95%: 0,15 - 0,36). Los autores reportan que las creencias sobre la medicación se correlacionaron significativamente con la adherencia al tratamiento.

De acuerdo con Horne R, et al(9) la valoración de las creencias sobre la medicación en los pacientes hipertensos a través de medidas válidas y confiables es fundamental para implementar intervenciones para mejorar la adherencia al tratamiento. La brevedad de las subescalas del *BMQ* para el tamizaje facilita su aplicación en la evaluación para su uso en la investigación y en la práctica clínica.

Las aplicaciones prácticas de la medición de las creencias sobre la medicación en pacientes con HTA el contexto colombiano, radica en que permite evaluar las creencias generales y específicas sobre la medicación antihipertensiva e identificar aspectos culturalmente relevantes para promover la adherencia terapéutica, teniendo en cuenta diferencias particulares y de grupos culturales, que pueden apoyar el desarrollo de intervenciones efectivas para contribuir al avance del conocimiento del fenómeno de estudio.

El presente estudio presenta algunas limitaciones. El *BMQ* fue administrado a una muestra de adultos hipertensos residentes en la región Caribe Colombiana. Se recomienda realizar estudios similares en otras regiones, teniendo en cuenta las diferencias culturales en cada una de las regiones de Colombia.

## Conclusiones

El *BMQ* versión español adaptado a la población hipertensa colombiana es un cuestionario que cuenta con evidencia de validez y confiabilidad para medir las creencias sobre la medicación en adultos con características similares a la muestra del estudio. Se recomienda realizar estudios de correlación entre creencias sobre la medicación con otras variables para realizar pruebas psicométricas adicionales como validez concurrente y validez predicativa.
